# Analysis of Australian Legislation Dealing with Contaminated Land

**DOI:** 10.1100/tsw.2002.185

**Published:** 2002-04-30

**Authors:** Steven Berveling

**Affiliations:** Abbott Tout Solicitors, Level 42, MLC Centre, 19-29 Martin Place, Sydney, NSW 2000, Australia

**Keywords:** legislation, drafting, contaminated land, ecologically sustainable development

## Abstract

An analysis of past and present Australian legislation for managing and regulating contaminated sites confirms that such legislation must deal with a number of specific matters.Australian legislation is effective in some of these areas but poor in relation to others. The analysis will assist other countries and jurisdictions to create effective legislation more speedily by avoiding identified problems.

## INTRODUCTION

A comprehensive analysis has been undertaken of the laws made by the different Australian states concerning contaminated land[2]. The aim of the analysis was to determine the effectiveness of the various laws and their provisions, so that a conclusion could be made about the most effective provision dealing with any particular aspect of contaminated land law.

Contaminated land has specific issues unique to it and not encountered by other environmental matters. These issues include the general locational stability of contamination and

1 General Counsel.

2 The Australian legislation concerning contaminated land that was examined includes: *Contaminated Land Management Act* 1997 (NSW); *Environmentally Hazardous Chemicals Act* 1985 (NSW); *Unhealthy Building Land Act* 1989 (NSW) *Contaminated Land Act* 1991 (Qld); *Environmental Protection Act* 1994 (Qld); *Environment Protection Act* 1993 (SA); *Environmental Protection Act* 1986 (WA); *Contaminated Sites Bill* 2000 (WA); *Environmental Protection Act* 1997 (ACT); *Environment Protection Act* 1970 (Vic); *Environmental Management and Pollution Control Act* 1994 (Tas).
Also considered (to a limited extent) were the *Environment Act* 1995 (UK), the *Soil Protection Act* 1994 (NL), and the *Comprehensive Environmental Response, Compensation and Liability Act* 1980 (U.S.) (CERCLA).
The current Australian legislation is available at www.austlii.edu.au/.

the difference in time between its causation and the acceptance that the contamination is a problem. These require management solutions not usually found in legislation dealing with other environmental matters.

The appropriateness of enacting contaminated land legislation is reflected in various international policy documents. A jurisdiction contemplating such legislation is greatly assisted by the Rio Declaration[3] and Agenda 21[4] to actually do so.

The Rio Declaration includes a number of principles relevant to contaminated land[5]. Chapter 20 of Agenda 21 refers to the rehabilitation of contaminated sites. It considers that the prevention of the generation of hazardous wastes and the rehabilitation of contaminated sites are key elements for the effective control of hazardous wastes[6]. It considers that controlling hazardous wastes is of paramount importance to ensure “proper health, environmental protection and natural resource management, and sustainable development”[7]. It recognises that both require knowledge, experienced people, facilities, financial resources, and technical and scientific capacities.

## ANALYSIS FINDINGS

The legislation of the various states dealing with the management and regulation of contaminated land all have some good aspects and some poor aspects. It is not possible to suggest the entirety of any particular act as an appropriate model for a jurisdiction contemplating legislation for the management and regulation of contaminated land. Instead, portions of the various acts may be able to be utilised as appropriate examples to deal with different aspects. How those various portions might deal with various issues concerning contaminated land is summarised below.

Whilst preferences can be suggested toward the legislative provisions of any one Act in relation to a particular issue dealing with contaminated land, it is important to ensure that any legislation created by amalgamating provisions from different Acts results in a unified whole, rather than in a disparate collection of disjointed and ill-fitting parts.

1. Principles of ecologically sustainable development, especially the principles of intergenerational equity and polluter pays, warrant legislation for the management of contaminated sites. International policy documents such as the Rio Declaration and Agenda 21 assist jurisdictions in justifying, if necessary, the need for such legislation.

The principle of intergenerational equity is inherent within legislation concerning contaminated land, as it provides for the maintenance and enhancement of the health, diversity, and productivity of the environment for the benefit of future generations. Therefore, the principle of intergenerational equity need not be articulated specifically in legislation concerning contaminated land. Nevertheless, doing so would serve as a reminder of one of its underlining reasons.

Whereas contaminated land legislation can seek to pursue the polluter-pays principle, this may not always be feasible in practice. A balance is required between it and other principles of ecologically sustainable development such as intergenerational equity. The polluter-pays principle does no more than seek to direct liability, without itself achieving something

3 United Nations Conference on Environment and Development, *Rio Declaration on Environment and Development* (“Rio Declaration”), UN Doc A/Conf.151/5/26 (12 August 1992).

4 United Nations Conference on Environment and Development, *Agenda 21*, (“Agenda 21”), UN Doc A/conf.151/26. http://www.un.org/esa/sustdev/agenda21text.htm. Accessed 4 June 2000 [Ed: accessed 27 February 2002], at chap. 20.

5 Rio Declaration, Principles 3, 11, 13, 15 and 16.

6 Note 4 at chap. 20.2.

7 Note 4 at chap. 20.1.

positive in the long term. In contrast, the principle of intergenerational equity seeks to achieve a benefit for many people in the future. The principle of intergenerational equity should therefore be favoured, by ensuring that action is undertaken with respect to contaminated land even if the polluter is not able to undertake that action.

2. The common law cannot deal effectively with the issue of contaminated sites. "Nuisance" is ineffective when it seeks to deal with contamination on the site where it was generated. It can only be used when transboundary migration of contamination is taking place, and even then the complainant needs to prove that the occupier of the site from which the contamination originates can (and should) stop the migration.

3. Subject-specific legislation is much more effective than generic legislation. Definitions in such legislation can be tailored specifically to the subject matter of contaminated sites. The definition of contaminated land determines how extensively the legislation can be applied. The definitions in any Act are crucial to its effective and desired functioning. Definitions in legislation concerning contaminated land should deal comprehensively with what contaminated land entails and should provide that any powers to issue orders or directives to take action are sufficiently wide to investigate or remediate it.

As is the case when drafting definitions in any legislation, great care must be taken so as to enable the legislation to be used comprehensively for its intended purpose. Also, care should be taken to ensure that any definitions are not surreptitiously modified by an *Interpretation Act*.

The legislation should deal with as many contaminated sites as possible. Given the underlying reason for the legislation (namely, to regulate and manage contaminated sites), it seems appropriate for its application to be determined by assessing the existence of *any* contamination on a given site. Thereafter, a qualitative assessment can be made, examining the extent and impact of the contamination. A priority list of contaminated sites can still be created if desired, as a result of which badly contaminated sites can be dealt with before those which present relatively little risk of harm.

4. Legislation concerning contaminated land is of little value if it does not require contaminated land to be identified. Such identification can be undertaken by a local council, as proposed by the U.K. legislation,[8] or by various members of the public, who can be obliged to notify the relevant authority of any knowledge concerning contaminated land.

It is important to determine when such notification is required to occur: should this happen upon suspicion or knowledge about *any* contamination on a site, or only when the notifier considers that the contamination presents a particular degree of risk of harm?

The Queensland legislation[9] deals best with this issue, by imposing upon an owner or occupier or local government authority a duty to notify the Environment Protection Authority (EPA) if aware that a notifiable activity[10] has been or is being carried on at the site, or that the land has been or is contaminated by some other means. It is then for the EPA to determine the extent and significance of the contamination.

8 *Environmental Protection Act* 1990 (U.K.) sec. 78B.
9 *Environmental Protection Act* 1994 (Qld) secs. 371, 372.
10 *Environmental Protection Act* 1994 (Qld) Schedule 2.

A shortcoming of the New South Wales and Australian Capital Territory legislation[11] in relation to the issue of notification is that the duty to report contaminated land is imposed only upon a person who considers that the land is contaminated in such a way as to present a significant risk of harm. Given the highly technical nature of the determination of contamination and the degree of harm that it might present, it is inappropriate to require members of the general public to ascertain whether the degree or extent of contamination is such that notification is required. Instead, a public authority with the necessary expertise should make that decision, as occurs in Queensland. Legislation should therefore require the notification of any contamination.

Lists assisting in the determination of the possibility of contamination have been prepared by various jurisdictions.[12] Such lists contain lists of activities that might cause contamination. They are of immense value, as they are usually the primary thought-triggering mechanism in ascertaining the possibility of contamination.

5. An objective of contaminated land legislation would be to return land to its previous uncontaminated state, so that it is available for future generations. Government departments should be given extensive powers to be able to order investigation and remediation as well as other activities. For that purpose, and because of the limited dictionary definitions of "remediation", it is necessary either to define comprehensively the range of activities that can be ordered, or else to include a general power (to order that whatever is considered appropriate in the circumstances be done).

The definitions of "remediation" in the New South Wales[13] and Australian Capital Territory[14] legislation are very comprehensive, by including the preparation of a long-term management plan (if any) for the land, the removal, dispersal, destruction, reduction, mitigation, or containment of contamination, and the elimination or reduction of any hazard from the contamination of the land (including the prevention of entry of persons on the land). They are also "inclusive", and examples of remediation activities not described in the legislation would nevertheless be included.

An order should, but might not, result in complete or appropriate remediation, or might lead to further action that is required. The legislation should therefore enable more than one order or directive to be issued with respect to any particular site and should also require that the work performed be audited by an accredited specialist.

Further, the relevant authority should be able to choose the most appropriate recipient of an order, as the identity or the location of all possible persons may not be known, and some of them may not have the funds to undertake the required works. The range of persons can be expressly delineated, as in the New South Wales *Contaminated Land Management Act* 1997,[15] or can be left up to the authority, as occurs (for example) in South Australia.[16]

11 *Contaminated Land Management Act* 1997 (NSW) s 60; *Environment Protection Act* 1997 (ACT) sec. 23A.

12 Environment Protection Authority of NSW and Department of Urban Affairs and Planning, *Managing Land Contamination: Planning Guidelines*, August 1998, Government Printer's Office, Sydney, Table 1 at 12; *Environmental Protection Act* 1994 (Qld) Schedule 2.

13 *Contaminated Land Management Act* 1997 (NSW) sec. 4.

14 *Environment Protection Act* 1997 (ACT) sec. 4.

15 *Contaminated Land Management Act* 1997 (NSW) sec. 12.

16 *Environmental Protection Act* 1993 (SA) sec. 93(2) provides that an environmental protection order must "specify the person to whom it is issued".

Some jurisdictions (e.g., New South Wales and Western Australia)[17] impose liability upon local authorities to be the recipients of an order or directive to take investigative or remedial action with respect to contaminated land, regardless of whether the local authority had any involvement with the contamination or the cause of the contamination. Queensland does so only if there is a suggestion of negligence by the local authority.[18] Whether to require a local authority to undertake such works is essentially a policy decision. If local authorities are considered appropriate recipients of orders, then the necessary funding should be made available to enable them to comply with such orders.

6. In order to be as complete as possible, publicly accessible databases should list known or suspected contaminated sites, rather than be records of orders or other actions that government authorities might have undertaken in relation to those sites.

Problems have occurred in the past with the entry of sites on a register of contaminated land. Whilst the register pursuant to the *Contaminated Land Act* 1991 (Queensland) was most comprehensive, its breadth and the large number of categories contained in it caused its demise. The public did not understand properly the differences between the categories and a stigma became attached to the entry of a site onto the register regardless of category.

The proposed legislation in Western Australia seeks to resolve the issue of stigma by limiting access to sites classified as "contaminated–remediation required" and "contaminated–restricted use". Access to other information on the database, which would include not contaminated, possibly contaminated, and decontaminated land, is limited to the owner or occupier of the site, a public authority, or a person prescribed as an interested person.[19]

Registers based on the argument that they should be limited to land that is known to be actually contaminated appear to be gaining ground over the second type of register, which includes possibly contaminated sites and remediated sites. This might be consistent with the suggestion in Agenda 21, which suggests only the need to establish and maintain inventories of, among other things, "contaminated sites that require rehabilitation".[20] However, limiting a register to land known to be actually contaminated, or to documents relating to remediation work prior to its completion, precludes future generations from collating all information about a particular site, including historical action that had been taken with respect to any contamination of the site.

7. Vendors should be responsible for providing prospective purchasers of land all knowledge held by them about contamination.

The Conveyancing Acts of the states impose obligations of varying intensities on vendors to disclose information. The New South Wales legislation requires that a vendor obtain a planning certificate from the local council, which certificate may indicate the council's extent of knowledge about contamination on the subject site.[21] However, a vendor would, in most cases, be more knowledgeable about that subject matter than a local council. It seems inappropriate to expose a third party to potential liability in negligence for providing advice

17 For example, *Contaminated Land Management Act* 1997 (NSW) secs. 12(5), 30; *Contaminated Sites Bill* 2000 (WA).
18 *Environmental Protection Act* 1994 (Qld) secs. 376, 391.
19 *Contaminated Sites Bill* 2000 (WA) at cl. 16.
20 Note 4 at chap. 20.22.
21 *Environmental Planning and Assessment Act* 1979 (NSW) s 149; *Conveyancing (Sale of Land) Regulation* 2000 (NSW) Item 1, Schedule 1.

used in a contract between other parties, one of whom should be more knowledgeable about the information sought.

The South Australian legislation deals best with the issue of advising purchasers. In South Australia, a vendor is required to serve upon a purchaser a statement setting out various matters,[22] including the existence of any environment protection order or any cleanup order registered in relation to the land.[23] The vendor is also required to advise whether any assessment has been undertaken to ascertain the presence of any contamination of the land, whether the vendor is aware of any manufacturing activities having occurred on the site, and whether any dangerous substances, chemicals, or fuels have been kept on the site.

8. Australia's legislation deals poorly with the exposure of lenders to liability concerning contaminated sites. The U.S. deals with the issue of lender liability by examining the extent of the lender's participation in the management of the facility on the contaminated site. The Australian acts have sought to replicate this criterion, but have not been successful in dealing effectively with the issue.

After the amendments in 1996 to CERLCA,[24] its provisions appear most appropriate in dealing with this complex issue, if only by reason of the numerous examples given in the Act as to when a lender might or might not be exposed to liability.[25] It seems unfortunate that the Australian states have not fully taken into account the problems that lenders had in relation to CERCLA and the modifications made thereto as a result.

A copy of the entire analysis is available on request. Please contact the author.

## BIOSKETCH

**Steven Berveling** is General Counsel, Abbott Tout Solicitors, Sydney, NSW, Australia. He is an environmental and pollution lawyer throughout Australia. Mr. Berveling holds B.Sc. and LL.B. degrees, and has submitted his S.J.D. thesis, a critique and comparative analysis of Australian legislation concerning contaminated sites.

22 Land and Business (Sale and Conveyancing) Act 1994 (SA), sec. 7.

23 Land and Business (Sale and Conveyancing) Regulation 1995 (SA), cl 7 and then items 53–56 in Division 1 of Table of Particulars of Schedule.

24 Asset Conservation, Lender Liability and Deposit Insurance Protection Act of 1996. PL 104-208, secs. 2502–05.

25 CERCLA, sec. 9601(20)(F).
